# Severe obesity, emotions and eating habits: a case-control study

**DOI:** 10.1186/s40608-016-0138-9

**Published:** 2017-01-07

**Authors:** M. Koski, H. Naukkarinen

**Affiliations:** 1 University of Helsinki, Helsinki, Finland; 2 Carea Hospital District, Kymenlaakso Psychiatric Hospital, University of Helsinki, Töölönkatu 26 C 55, 00260 Helsinki, Finland

**Keywords:** Eating habits, Emotions, Body mass index (BMI), Overweight, Severe obesity

## Abstract

**Background:**

Obesity has a multifaceted etiology that involves genetic, biological and behavioral factors, body growth, eating habits, energy expenditure and the function of adipose tissue. The present study aimed to expand upon knowledge about the relationships among obesity, emotions and eating habits in severely obese individuals using a case-control method.

**Methods:**

The subject group consisted of 112 individuals (81 females and 31 males) receiving a permanent disability pension primarily for obesity. The control subjects were randomly selected from the same area and were receiving a disability pension for a different primary illness. The controls were matched with the subjects by the place of residence, sex, age, the time since the pension was granted and occupation. Psychiatric interviews were conducted on all participants. The results were analyzed using the chi-squared test (χ^2^-test) and the percent distribution. The subject and control groups were compared using the t-test for paired variables. Conditional logistic regression analysis was also conducted.

**Results:**

The emotional state of eating was significantly associated with quarrels and feelings of loneliness. The subjects suffered from night eating syndrome, which was associated with an increased risk of early retirement. Binge eating syndrome was observed more frequently in the study group. The subjects reported feeling increased hunger compared with the controls. A significant percentage of the subjects had a body mass index of ≥ 40. No differences in eating habits were observed between the groups.

**Conclusion:**

This study provides information on the relationship between emotions and eating habits in obesity, which is a rarely studied topic. We believe that our study provides a novel and necessary overview of the associations among severe obesity, emotions and eating habits.

## Background

Obesity has a multifaceted etiology that includes genetic, biological and behavioral factors, such as body growth, eating habits, energy expenditure and the function of adipose tissue. Recently, there has been growing research interest in obesity. Studies have explored the psychological significance of food in individuals who struggle with weight. Furthermore, the possibility of an emotional disorder secondary to obesity should always be evaluated separately.

### Biological aspects of eating habits

Many peripheral hormones control appetite and food intake. Hunger and satiety hormonal signals are released from the adipose tissue, pancreas and gastrointestinal tract and travel to the brain [1, 2]. Leptin is synthesized by adipose tissue and decreases the impact of appetite-stimulating neuropeptides [3]. Insulin is a pancreatic hormone that maintains glucose homeostasis. Like leptin, insulin is capable of modifying the dopaminergic pathway and can influence eating behaviors [4, 5]. Ghrelin is produced by the stomach and increases food intake [6]. Peptide tyrosine-tyrosine (PYY) decreases appetite and increases satiety [7, 8]. Glucagon-like peptide decreases food intake [9]. Cholecystokinin (CCK) helps to control appetite, ingestive behavior and gastric emptying [10]. Neuroimaging studies, which have assessed appetite and body weight regulation, have found modifications in dopaminergic function in response to eating or food cues [11, 12].

### Psychological aspects of eating habits

Hudson and Williams [13] found that eating in obese individuals was more frequently associated with the emotions of anger, boredom and depression compared with normal-weight individuals. Eating alone was also much more common among the obese individuals, and they often tried to conceal their eating [13].

According to Hamburger [14], hyperphagia may involve eating to relieve emotional tension, such as that caused by unspecified anxiety or feelings of rejection. Masheb and Grilo [15] examined emotional overeating in overweight patients with binge eating disorder (BED) and found significant correlations between the emotional overeating items and total score and binge frequency, eating disorder features and depressive symptomatology. Further, Yannakoulia et al. [16] have found that the dietary patterns differ between anxious men and women, after adjusting for potential confounders.

According to Nedeltcheva et al. [17], recurrent bedtime restriction can modify the amount, composition and distribution of human food intake. Sleeping short hours in an obesity-promoting environment may facilitate the excessive consumption of energy from snacks but not from actual meals.

According to Ostrovsky et al., obesity in males and females is associated with binge eating, social anxiety and emotional eating [18]. The findings of Dalton et al. provide additional support that the trait of binge eating represents a hedonic subgroup of obesity. The authors have emphasized the importance of food composition and have determined that gluttons fail to recognize when they are full [19]. Dalle et al. have investigated personality features that influence eating habits, the development of obesity and the likelihood of treatment success for obesity and have identified particular personality traits (binge eating and night eating) that are associated with obesity in women [20]. One study has shown that obese individuals with BED represent a specific subgroup of obesity with increased food-related impulsivity. In addition, the authors found increased reward sensitivity in obese individuals, which was more pronounced among those with BED [21]. Lent et al. have identified similarities between addictive personality and poor eating habits [22]. Further, a lack of self-discipline has been demonstrated to be highly associated with BED and obesity [23].

Considering the background information presented above, this study examined the relationships among severe obesity, emotions and eating habits; these associations have not been fully investigated to date. In this case-control study, we assessed emotions and eating habits in a group of severely obese individuals.

## Methods

### The subject and control groups

The subjects consisted of 152 individuals living in southern Finland who were receiving a permanent disability pension primarily for obesity. Among these individuals, 19 had been granted a temporary pension and were excluded from the sample. In addition, individuals who had died or who no longer received a pension were also excluded from the sample. The subject group consisted of 112 patients (81 females and 31 males). The control subjects were randomly selected from the same area and were receiving a disability pension for a different primary illness. The controls were matched with the subjects by place of residence and sex. In addition, all steps were taken to match the controls based on age, the time since the pension was granted and occupation. The occupations of the controls were either the same as the subjects or unknown.

The male and the female controls were selected separately. Three controls for each female subject and five for each male subject were selected from among the potential controls. For the interview, attempts were made to have at least two controls for each female subject and three for each male subject. In total, 510 participants (112 subjects and 398 controls) were enrolled in this study. More males than females refused to participate in this study. In addition, more matched controls than subjects refused to participate. The numbers of females and males are provided in Table 1. The data were collected in cooperation with the Social Insurance Institution of Finland. Three letters of invitation to participate were sent to each subject and control. The letters were discreetly worded and emphasized the confidentiality of the study. Most of the individuals who refused to participate indicated their reasons for refusal in writing, and this information was made available to the authors. The interviews were blinded. The interviewer did not know whether the participant was in the subject or control group until all of the research material was compiled and the codes were revealed.

**Table 1:** Basic characteristics of study participants

	Study group	Control group	Significance (χ^2^-test)
Subject status	112	262	
• No answer	5 (m = 1, f = 4)	22 (m = 8, f = 14)	
• Dropped out	37 (m = 9, f = 28)	61 (m = 18, f = 43)	
• Agreed	75 (m = 22, f = 53)	179 (m = 67, f = 112)	
Age during psychiatric examination			
• 20-24	-	0.6%	
• 25-29	1.4%	0.6%	
• 30-34	-	0.6%	
• 35-39			
• 40-44	-	1.2%	
• 45-49	4.3%	2.9%	
• 50-54	14.5%	9.4%	
• 55-59	31.9%	27.1%	
• 60-64	43.5%	48.8%	
• 65-69	4.3%	8.8%	
BMI			
• ≤24.9	1.3%	34.3%	
• 25.0-29.9	4.0%	47.3%	
• 30.0-34.9	18.7%	14.2%	
• 35.0-39.9	37.3%	3.6%	
• 40.0≥	38.7%	0.6%	
Marital status			p = 0.0894
• Unmarried	10.7%	15.7%	
• Married	62.7%	59.6%	
• Widowed	14.7%	13.5%	
• Divorced	6.7%	10.7%	
• Common-law marriage	5.3%	0.6%	
Basic education			p = 0.2457
• Primary school	89.3%	90.4%	
• Lower secondary school	6.7%	2.2%	
• High school	-	2.2%	
• Other	4%	3.9%	
Occupational category	n = 22 (m) n = 53 (f)	n = 66 (m) n = 112 (f)	p = 0.901 (m) p = 0.5930 (f)
• Technical, scientific, sociological, and artistic work	m = 0% f = 0% total = 0%	m = 0% f = 4.5% total = 2.2%	
• Accounting and clerical work	m = 4.5% f = 5.7% total = 5.1%	m = 1.5% f = 2.7% total = 2.1%	
• Commercial work	m = 4.5% f = 17.0% total = 10.8%	m = 4.5% f = 10.7% total = 7.6%	
• Agricultural, forestry, and fishing	m = 0% f = 7.5% total = 3.7%	m = 3.0% f = 7.1% total = 5.1%	
• Transport and communication work	m = 27.3% f = 7.5% total = 17.4%	m = 24.2% f = 4.5% total = 14.3%	
• Industrial work	m = 50.1% f = 17.0% total = 33.5%	m = 48.6% f = 21.4% total = 35.0%	
• Service work	m = 13.6% f = 45.3% total = 29.5%	m = 18.2% f = 49.1% total = 33.7%	
• Total	m = 100% f = 100% total = 100%	m = 100% f = 100% total = 100%	
Social classification	• According to Bruun’s social classification		p = 0.050 (m) p = 0.936 (f)
• I = First social class	4.2%	2.3%	
• II = Second social class	12.5%	17.7%	
• IIII = Third social class	50.0%	57.7%	
• IV = Fourth social class	33.3%	22.3%	

*m* male

*f* female

The participants were interviewed by the author of this manuscript using an interview form that was partially filled out during the interview and then completed after. The pilot study (*n* = 30) was conducted at the neurological ward of Hesperia Hospital in Helsinki, Finland. This ward contained neurological patients with commonly observed diseases. Adjustments were made to the interview form following the pilot study based on how well the participants understood the form and the amount of time needed to fill it out.

Body mass index (BMI) was calculated using the following formula: body weight (kg) divided by the square of body height. According to the World Health Organization (WHO) guidelines, the weights were classified as follows: overweight, BMI 25 ≤ 29; obese, BMI 30 ≤ 34; severely obese, BMI 35 ≤ 4; and morbidly obese, BMI > 40.

The Sickness Insurance Act and the National Pensions Act provide insurance against disability for all residents of Finland. The National Pensions Scheme offers basic pension insurance to all Finnish citizens. Age, professional skills, and other factors are also important for evaluating disability. Individual differences in working capacity should be recognized, with consideration of the applicants’ ages.

Further, standard occupational classifications from the Social Insurance Institution (1982) were used in this study.

The study protocol was approved by the Ethics Committee of Hesperia/Aurora Hospital (a community psychiatric hospital in Helsinki) and Lapinlahti Hospital (a psychiatric clinic at Helsinki University)/Psychiatric Centrum of Helsinki University. Informed consent was obtained from the participants, and the ethical principles of the Declaration of Helsinki were followed throughout the study.

### Statistical methods

The results were analyzed using the χ^2^-test, t-test and conditional logistic linear regression analysis. Because the subjects were matched, the means were calculated for both the subjects and controls, and then the data were analyzed using the t-test for paired variables. Paired variables that were statistically significant were further analyzed by conditional logistic regression analysis. For the results that remained significant, the risk ratio (RR) and the upper and lower limits of the confidence interval were calculated. Statistical analysis was performed using Statistical Package for Social Sciences software (SPSS), version 11.01 (Windows, Chicago, IL, USA). Logistic linear regression analysis was performed using GLIM program [24]. For continuous variables, the results were analyzed using the paired t-test. Conditional logistic regression is a straightforward analysis provided that the data are grouped separately for each individual. A major advantage of this technique is that it is easy to perform and has inherent flexibility when all data for each individual are included in analysis.

The observations in each matched set included one case and 0-5 controls. These observations were each considered a count in logistic regression analysis, and the model included a Poisson error distribution and logarithmic link function; therefore, the model was a special form of a log-linear model. The linear predictor in the systematic part of the model for each observation is a linear function of the observed exposure variables for each individual plus a constant (set) term, which may vary from matched set to matched set. This model for analysis of case-control data is termed a conditional logistic regression model.

In statistical analysis, for cases in which none of the controls completely matched the subject, the next most closely matched control was used to avoid decreasing the size of the subject group. Use of this matched control approach resulted in exclusion of some of the subjects who had agreed to participate in the study during statistical analysis because no matched control was available. Because several specific variables were absent in some cases, the number of observations available for comparisons was further diminished [25].

Group differences were considered highly significant, significant, and almost significant when the probabilities (p) of error in rejecting the null hypothesis were *p* < 0.001, *p* < 0.01, and *p* < 0.05, respectively.

### Study participation refusal

In total, 37 individuals refused to participate (9 males and 28 females) in the study. One male subject could not be contacted after initial inclusion in the study, and one female subject dropped out of the study before the psychological test was administered. The mean ages of the refused male and female participants in the study group were 59 (standard deviation (SD), 3.61) and 61 (SD, 4.46) years, respectively. A total of 31 participants had primary school education, and 34 had no vocational education. The individuals who refused to participate had the same education level, age and sex distribution as the participating individuals (Table 1). More matched controls than subjects refused to participate in this study. Table 1 shows the complete information for the participants in this study.

## Results

Table 1 illustrates the background characteristics of the study participants.

The mean weight of the subjects (*n* = 75) was 106.2 kg (SD = 18 kg), and that of the controls was 72.3 kg (SD = 14.3 kg). Matching of the subjects and controls was successful. The χ^2^-test revealed that there were no significant differences in age, marital status, basic education level or occupation between the subjects and controls. At the time of the personal interview, 40% of the female subjects and none of the female controls had a BMI of over 40 kg/m^2^, and 33% of the female subjects had a BMI of 35-40 kg/m^2^. Among the men, 36% of the subjects and none of the controls had a BMI exceeding 40 kg/m^2^, and 41% of the subjects and 2% of the controls had a BMI of 35-40 kg/m^2^. In addition, 6% of the female subjects and none of the male subjects had a BMI of 25-30 kg/m^2^.

**Fig. 1:**
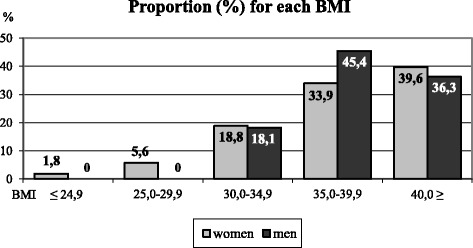
Body mass index distribution for the participants in the study group

Among the subjects, 91% (68 subjects) had received a secondary somatic diagnosis from the Social Insurance Institution. The most common secondary diagnosis was “diseases of the musculoskeletal system and connective tissue”, which had been diagnosed in 38% of the case subjects. Among the controls, “disease pertaining to the cardiovascular organs” was the primary diagnosis (20% of the controls). All of the controls had been diagnosed with a primary illness other than obesity (Table 1 and Fig. 1).

Table 2 shows the influence of the emotional state on eating. The results showed that 14% of the subjects and 23% of the controls reported eating following a quarrel. The paired t-test showed that this difference was significant (*p* = 0.007), and logistic regression analysis revealed a risk ratio of 45 and confidence interval of 14-145; in addition, the χ^2^-test revealed that this difference was highly significant (*p* = 0.001). Among the subjects, 3% reported eating when they were angry, and this behavior was not observed among the controls. Similar results were obtained with the paired t-test (*p* = 0.159) and χ^2^-test (*p* = 0.086), which showed non-significant differences between the groups. In addition, 4% of the subjects and 1% of the controls reported eating when they felt displeasure. Similar results were obtained with both the paired t-test (p = 0.083) and χ^2^-test (*p* = 0.154), which showed non-significant differences between the groups. Furthermore, 15% of the subjects and 7% of the controls reported eating when feeling pleasure; the paired t-test (*p* = 0.073) and χ^2^-test (*p* = 0.054) showed that this difference was almost significant. Among the controls, 1% reported eating when excited; the paired t-test (*p* = 0.182) and χ^2^-test (*p* = 0.557) again revealed non-significant differences between the groups. Moreover, 11% of the subjects and 3% of the controls reported eating when feeling lonely; this difference was also almost significant according to the paired t-test (*p* = 0.197) and χ^2^-test (*p* = 0.023). Finally, 18% of the subjects and 12% of the controls said that their eating was associated with some non-specific emotional state (paired t-test; *p* = 0.211; and χ^2^-test; *p* = 0.311).

**Table 2:** Influences of different emotional states on eating

Statistical significance					
Eating association with		Study group	Control group	Paired t-test	Fisher's exact test
- quarrelling	Yes	14%	2%	(p = 0.007)	(p = 0.001)
	No	86%	98%		
total		74	175		
- anger	Yes	3%	0%	(p = 0.159)	(p = 0.086)
	No	97%	100%		
total		73	175		
- displeasure	Yes	4%	1%	(p = 0.083)	(p = 0.154)
	No	96%	99%		
total		73	175		
- pleasure	Yes	15%	7%	(p = 0.073)	(p = 0.054)
	No	85%	93%		
total		73	175		
- excitement	Yes	0%	2%	(p = 0.182)	(p = 0.557)
	No	100%	98%		
total		73	175		
- loneliness	Yes	11%	3%	(p = 0.197)	(p = 0.023)
	No	89%	97%		
total		73	175		
- non-specific emotional state	Yes	18%	12%	(p = 0.211)	(p = 0.311)
	No	82%	88%		
total		73	173		

We also investigated the prevalence of binge eating and found that 8% of the subjects and 2% of the controls had experienced periods of binge eating, although this difference was not significant (*p* = 0.060). Furthermore, 36% of the subjects and 11% of the controls reported having night eating syndrome (NES). Logistic regression analysis revealed that the subjects with NES had a significantly higher risk of early retirement (being placed on pension early) (RR: 4.5, confidence interval: 2.5-8.1, *p* = 0.000).

In addition, 15% of the subjects and 3% of the controls reported being constantly hungry, while 20% of the subjects and 24% of the controls said that they were often hungry (paired t-test, *p* = 0.039). Logistic regression analysis revealed that these differences were almost significant, and the χ2-test indicated significant differences (*p* = 0.008).

The respondents were also asked when they felt hungry, and 34% of the subjects and 27% of the controls reported being hungriest in the evening, suggesting that most of their eating took place in the evening. Furthermore, 10% of the subjects and 3% of the controls said that they were the hungriest at night, whereas 10% of the subjects and 15% of the controls reported being hungry in the morning. The differences in feelings of hunger approached statistical significance between the subjects and controls in the paired t-test (*p* = 0.021), and the χ^2^-test indicated significant differences (*p* = 0.004).

To determine the respondents’ eating habits, they were asked which foods they liked and disliked. The results showed that 54% and 52% of the subjects and controls, respectively, reported eating all types of food. Only 9% of both the subjects and controls reported liking vegetables. None of the subjects and 1.8% of the controls reported eating fruits and berries. None of the subjects liked sausage, although 30% liked meat, compared with 31% of the controls (χ^2^-test, *p* = 0.856). The eating habit findings were similar between the males and females.

Overall, 40% of the subjects and 41% of the controls reported liking sweets (χ^2^-test, *p* = 0.887).

Table 3 shows family attitudes toward food and eating during the respondents’ formative years. The results showed that 6.7% of the subjects and 6.6% of the controls were taught that eating was very important during the formative years. In addition, 2.7% of the subjects stated that everything on the plate had to be eaten during the formative years. Moreover, 5.3% of the subjects and 5.1% of the controls only provided the different courses they ate during a meal. The subjects reported that their mothers had prepared their meals (χ^2^-test; *p* = 0.85).

**Table 3:** Family attitudes toward food and eating during the respondents’ adolescence

Description	Study group	Control group	Total	Significance χ^2^ = 0.85
Nothing in particular	58.7%	57.6%	57.9%	
An important occasion	6.7%	6.8%	6.7%	
Marked by scarcity	16.0%	16.9%	16.7%	
The respondent listed only the courses	5.3%	5.1%	5.2%	
A feeling of emptiness	2.7%	2.8%	2.8%	
Other	8.0%	9.0%	8.7%	
Everything on the plate had to be eaten	2.7%	1.7%	2.0%	
Total%	29.8%	70.2%	100.0%	

The respondents were further asked who cooks in their present family, and 75% of the subjects and 72% of the controls reported that they did the cooking themselves, whereas 22.7% of the subjects and 23.6% of the controls stated that their spouse made dinner. Finally, 96% of both the subjects and controls reported eating mostly at home.

To obtain information about the development of obesity, the participants were asked about their own perceptions of why they were obese. The results showed that 17% of the subjects and 3% of the controls indicated that metabolic factors were the reason for their obesity. Additionally, 42% of the participants thought that overeating caused their obesity, and 18% believed that they were overweight due to lack of exercise. Furthermore, 5% of the subjects felt that they were not excessively overweight, whereas 26% of the controls reported similar feelings (χ^2^-test, *p* = 0.0007). Most notably, 6% of the males and 4.7% of the females in the subject group believed they did not have obesity. The χ^2^-test showed that this difference approached significance among the men (*p* = 0.022); however, the χ^2^-test revealed a significant difference between the subject and control groups (*p* = 0.004) (Table 4).

**Table 4:** The reasons given by the participants for their obesity

	Study group n = 60	Control group n = 102	Significance χ^2^ = 0.0007
Metabolism	16.7%	2.9%	
Nothing to do with food	18.3%	10.8%	
Eating too much	41.7%	42.2%	
Exercise too little	18.3%	18.6%	
Not overweight	5.0%	25.5%	
Total %	100	100	

Among the participants, 16% reported feeling angry when they attempted to lose weight, 10% reported feeling tired, 7% stated that they thought only of eating, 11% reported feeling good, 8% reported feeling weak and 4% reported feeling stressed. In addition, 3% of the subjects stated that trying to lose weight made them feel depressed, which was not reported by any of the controls.

## Discussion

The results of this study demonstrated that the emotional state was significantly connected to eating in association with quarrels and loneliness. In addition, feelings of anger and pleasure were also related to eating habits. BED was more common in the subject group than in the control group in this study. Logistic regression analysis revealed that the subjects with NES had a significantly higher risk of early retirement because of obesity.

A significant difference was observed between the subject and control groups in the feeling of hunger, with the subject group experiencing increased hunger. Further, the subjects were hungrier more often during the evening and night compared with the controls.

We found minor differences between the subject and control groups in their responses to questions about foods that they liked or disliked. Surprisingly, there was no significant difference in the preference for sweets between the subject and control groups.

In this study, we also investigated eating habits during the formative years. The majority of the subjects reported that everything on their plate had to be eaten. In their present family, many of the participants reported eating mostly at home and that they did the cooking themselves. These findings were similar between the subject and control groups.

When the participants were asked about their own perceptions of their obesity status, few of the subjects felt that they were not excessively overweight, whereas one-quarter of the controls reported having similar feelings. This finding was statistically significant.

Bruch [26] has reported that the feeling of hunger is not innate and that it is somewhat acquired by learning. In overeating disorders, the feeling of hunger is abnormally enhanced, prompting the urge to eat. The feeling of hunger gets mixed with other signals of discomfort and emotional tension. Individuals eat when they are disappointed, and they use their love of eating to compensate for these feelings. Bruch has also discussed “reactive obesity”, which affects individuals who eat when they are feeling tension, anxiety or loneliness. According to Hamburger (14), overeating tends to be associated with very strong emotional feelings; individuals eat when they are emotional disturbed.

Obesity is associated with uncontrolled hunger, anger, anxiety, boredom and fatigue. Varsha et al. [27] have also demonstrated that obese individuals have poor control of eating; they eat when they have stress, anxiety and boredom. Hudson and Williams [13] reported similar findings. According to Rosenthal and Wehr [28], who studied “seasonal affective disorder,” vegetative symptoms increase hunger and weight gain.

In this study of severe obesity, emotions and eating habits, we also found a connection between eating habits and emotions.

We found that loneliness was the emotion most strongly associated with eating. Brownell and Wadden have found that many individuals use food to escape and that they may use food as a substitute for relationships. Many obese individuals report that food is their best friend, and they look forward to times when they can be alone with food [29].

Gearhardt et al. studied the eating habits of patients with BED and found that nearly half of the patients had a food addiction. In addition, they detected significant associations between negative affect and emotional dysregulation, eating disorders, psychopathology and low self-esteem in the BED patients [30]. The number of binge eaters in the present study was lower compared with previous studies [31] [32] [33]. In addition, the prevalence of NES in the present study was higher than that reported by Stunkard et al. [34]. Marcus et al., who investigated obesity in nurses, found that the severity of binge eating was increased in younger individuals and in individuals with higher levels of obesity. In addition, the severity of binge eating has been shown to be related to dietary restraint [35]. According to Napolitano et al. [36], NES is a subcategory of obesity that overlaps with binge eating. In addition, Pawlow et al. have found that stress and anxiety play roles in NES and have suggested that practicing relaxation techniques may be an important component of treatment of this condition [37]. Further, our findings are in line with those of Masheb and Grilo [15]; however, we could not directly compare the findings of that study with our results because that group studied BED patients, only some of whom were overweight.

We found that the obese individuals in the subject group experienced and reported feeling hunger more often than the individuals in the control group; this difference approached statistical significance. Our findings are in contrast with those of Varsha et al., who have found that although obese patients report having enormous appetites, they are able to consume a large amount of food before they feel full. Further, they have found that individuals with obesity rarely report feelings of hunger [27].

Konttinen has investigated uncontrolled and emotional eating among Finnish men and has shown that individuals who are motivated to lose weight eat less [38]. In addition, Konttinen has found that emotional eating and depressive symptoms are correlated with increased weight in both males and females. Furthermore, emotional eating has been shown to be related to eating sweets in both genders, and depressive symptoms and non-emotional eating have been demonstrated to be related to reduced consumption of fruits and vegetables. These findings support the associations of emotional eating and depressive symptoms with eating unhealthy food [39]. In our study, the same amount of subjects and controls reported liking to eat sweets. We did not find any difference in the consumption of fruits and vegetables between the groups.

Eating habits are culturally dependent and are learned as a child. In addition to the quality of nutrition, more attention should be paid to the emotional reasons for eating, as suggested by Brownell and Wadden [29]. In this study, we assessed childhood eating habits and found minor differences between the study and control groups.

The clear advantage of this study is its use of a non-selective sample of individuals with severe obesity. Unlike most studies of obesity, the subjects were not recruited from a group of dieters. This study concentrated on a group of individuals receiving a disability pension for obesity. All of the subjects were individually interviewed by an experienced psychiatrist. The interview was conducted such that the interviewer did not know whether the individual was in the subject or control group. The subject and control groups were successfully matched. The occupational and social statuses were nearly identical between the two groups. Both the subjects and controls were receiving a pension for the same duration of time, which minimized influences of the subjects’ living situations. The fact that the controls were selected by random sampling using data from the Social Insurance Institution of Finland adds further value to our findings. This study was conducted by psychiatrists; although additional benefits would have been achieved by performing analyses with the expertise of a dietician, this was not possible in this study.

## Conclusion

We believe that our study provides a novel and necessary overview of severe obesity, emotions and eating habits. We hope that this overview will provide insights that will help to revise and update the current knowledge on obesity. Our finding of a connection between emotions and obesity confirms the importance our study. We believe that this study provides encouraging possibilities for research on the potential health effects of severe obesity and it’s development.

## Abbreviations

BED: Binge eating disorder
BMI: Body mass index
CCK: Cholecystokinin
NES: Night eating syndrome
PYY: Peptide tyrosine-tyrosine
SPSS: Statistical Package for Social Sciences Software
WHO: World Health Organization
x^2^: Chi-Squared Test
